# Cytoarchitectonic mapping of the human brain cerebellar nuclei in stereotaxic space and delineation of their co-activation patterns

**DOI:** 10.3389/fnana.2015.00054

**Published:** 2015-05-13

**Authors:** Stefanie Tellmann, Sebastian Bludau, Simon Eickhoff, Hartmut Mohlberg, Martina Minnerop, Katrin Amunts

**Affiliations:** ^1^Department of Psychiatry, Psychotherapy and Psychosomatics, RWTH Aachen University and JARA-BrainAachen, Germany; ^2^Institute of Neuroscience and Medicine (INM-1), Structural and Functional Organization of the Human Brain, Research Centre JülichJülich, Germany; ^3^Institute for Clinical Neuroscience and Medical Psychology, Heinrich Heine UniversityDüsseldorf, Germany; ^4^Cécile and Oskar Vogt Institute of Brain Research, Heinrich Heine UniversityDüsseldorf, Germany

**Keywords:** cytoarchitecture, cerebellar nuclei, brain mapping, human brain atlas, SPM Anatomy Toolbox

## Abstract

The cerebellar nuclei are involved in several brain functions, including the modulation of motor and cognitive performance. To differentiate their participation in these functions, and to analyze their changes in neurodegenerative and other diseases as revealed by neuroimaging, stereotaxic maps are necessary. These maps reflect the complex spatial structure of cerebellar nuclei with adequate spatial resolution and detail. Here we report on the cytoarchitecture of the dentate, interposed (emboliform and globose) and fastigial nuclei, and introduce 3D probability maps in stereotaxic MNI-Colin27 space as a prerequisite for subsequent meta-analysis of their functional involvement. Histological sections of 10 human *post mortem* brains were therefore examined. Differences in cell density were measured and used to distinguish a dorsal from a ventral part of the dentate nucleus. Probabilistic maps were calculated, which indicate the position and extent of the nuclei in 3D-space, while considering their intersubject variability. The maps of the interposed and the dentate nuclei differed with respect to their interaction patterns and functions based on meta-analytic connectivity modeling and quantitative functional decoding, respectively. For the dentate nucleus, significant (*p* < 0.05) co-activations were observed with thalamus, supplementary motor area (SMA), putamen, BA 44 of Broca’s region, areas of superior and inferior parietal cortex, and the superior frontal gyrus (SFG). In contrast, the interposed nucleus showed more limited co-activations with SMA, area 44, putamen, and SFG. Thus, the new stereotaxic maps contribute to analyze structure and function of the cerebellum. These maps can be used for anatomically reliable and precise identification of degenerative alteration in MRI-data of patients who suffer from various cerebellar diseases.

## Introduction

The cerebellar nuclei show a complex morphology and their full extent is partly invisible in routine Magnetic Resonance Imaging (MRI). Recently, a MRI-based atlas (SUIT) of the three parted cerebellar nuclei (dentate, interposed, and fastigial), which is based on 7T MR images of 23 subjects, has been introduced (Diedrichsen et al., 2011). Its spatial resolution is 0.5 mm. This resolution, however, does not enable to identify cellular details as obtained in histological mapping in cell-body stained sections. Such detailed maps could be beneficial for an anatomical reliable and precise identification of activation foci obtained in neuroimaging experiment, or degenerative alteration in MRI-data of patients who suffer from various cerebellar diseases.

Impairment of the cerebellum and its output pathways can lead to several clinical syndromes, e.g., cerebellar ataxia (Manto, 2002). Neurodegeneration, targeting within the cerebellum especially the dentate nucleus, occurs, e.g., in an autosomal dominant inherited disorder, called Spinocerebellar Ataxia Type 3 (SCA3; Rub et al., 2008, 2013; Scherzed et al., 2012). However, these neuropathologically observed changes of the dentate nuclei in SCA3 have yet not been demonstrated by imaging techniques *in vivo*, most probably due to the spatial resolution.

The four cerebellar nuclei, i.e., the dentate (DN), emboliform (EN), globose (GN), and fastigial nucleus (FN), are located in the depth of the cerebellar hemispheres in close vicinity to the fourth ventricle. The most laterally located dentate nucleus appears as a convoluted band containing rounded large multipolar neurons. It is the largest nucleus and well visible in routine MR images. A partition of the human dentate nucleus into a dorsal micro- and a ventral macrogyric part has been mentioned in an early description of the cerebellar nuclei (Stilling, 1864). Since then, this subdivision has been repeatedly replicated both in early (Winkler, 1926; Vogt and Vogt, 1942; Hassler, 1950; Fix and Treff, 1970) and more recent (Arras, 1987; Voogd, 2003; Deoni and Catani, 2007) studies. It was also reported that the ventral part contains more iron than the dorsal one (Gans, 1924), which may indicate an increased vulnerability for degenerative disorders (e.g., Schulz and Pandolfo, 2013). A similar dorsal–ventral subdivision of the dentate nucleus was also shown in primates by using invasive tracing (Dum and Strick, 2003).

In contrast to the dentate nucleus, the interposed nucleus – the wedge-shaped emboliform nucleus and the more rounded globose nucleus – are found within the paravermal region, next to the medial border of the dentate nucleus, and close to the dentate hilus. The fastigial nucleus, also known as tegmental nucleus, is the most medially located cerebellar nucleus and builds the roof of the fourth ventricle (e.g., Kozlova, 1984; Nieuwenhuys et al., 2008). Each nucleus receives inhibitory afferents from distinct parts of the ipsilateral cerebellar cortex. Large parts of the cerebellar cortex project to the dentate nuclei of both hemispheres. The interposed nuclei receive information from the paravermal zone, and the fastigial nucleus from the vermal cerebellar cortex as well as from the flocculus (Manto, 2002).

All cerebellar nuclei are interconnected with the rest of the brain through the cerebellar peduncles. The middle cerebellar peduncle relays information from the cerebral cortex via the pontine nuclei to cerebellar structures. Efferent fibers of the dentate and interposed nuclei reach, via the superior peduncle, thalamic nuclei, and sensorimotor areas (Carpenter, 1991; Manto, 2002; Dum and Strick, 2003). Further efferents from the dentate nucleus project to the red nucleus and subsequently to the inferior olives; the latter in turn project back to the dentate nucleus, forming the *Guillain-Mollaret-Triangle* (Lavezzi et al., 2009). The fastigial nucleus sends projections through the inferior peduncle to the vestibular nuclei and the reticular formation. A few fibers depart from the cerebellar uncinate fascicle and ascend to thalamic subnuclei VLc and VPLo (Carpenter, 1991).

The vascular network forms another aspect of cerebellar organization. The cerebellar nuclei are supplied by the rhomboidal artery, a branch of the superior cerebellar artery (Icardo et al., 1982). It runs in parallel to the superior cerebellar peduncle. When the hilum of the dentate nucleus is reached, the rhomboidal artery divides into a network of smaller vessels, the arcuate arterioles, showing a precise vascular pattern, and building anastomoses with cortical branches from the posterior inferior cerebellar artery (Icardo et al., 1982). The veins of the dentate nucleus are composed of several veins draining its external surface (into the venous star and the cortex-perforating veins) and one single vein draining its internal surface, emerging from the hilum of the dentate nucleus, and running along the superior cerebellar peduncle to the precentral cerebellar vein (Tschabitscher and Perneczky, 1976; Tschabitscher, 1979; Di Ieva et al., 2011).

The role of mammalian cerebellar nuclei in motor functions has been described in detail (Jansen and Brodal, 1942; Chambers and Sprague, 1955; Jansen et al., 1958), but in accordance to more recent studies the cerebellar nuclei – especially the dentate nucleus – are not only involved in modulation of movements but also in cognition (Dum and Strick, 2003; Schmahmann and Caplan, 2006; Schmahmann, 2010; Kuper et al., 2011a, 2012; Timmann, 2012). The dorsal part of the dentate nucleus is supposed to be responsible for motor performance whereas a ventral part was identified as cognitive or non-motor part. Assuming a functional subdivision of the cerebellar nuclei (Manto, 2002; Timmann et al., 2003), it was postulated that certain nuclei or subdivisions of a nucleus are involved in a specific task of cognition and even emotion (see also Gerwig et al., 2003; Maschke et al., 2003; McNaughton et al., 2004; Kuper et al., 2013). For example it has been shown that the fastigial and interposed nuclei take part in conditioning (Timmann, 2012). The dentate nucleus, regarded as the phylogenetic highest developed cerebellar nucleus in humans (e.g., Mihajlovic and Zecevic, 1986; O’Rahilly and Müller, 2006), seems to be involved in speech or cognitive–associative learning (Thurling et al., 2011).

Several studies reported data regarding volumes, cell densities, and cell sizes of the cerebellar nuclei in humans (cf. Kölliker, 1889; Lugaro, 1895; Cajal and Santiago, 1953; Braak and Braak, 1983; Kozlova, 1984; Mihajlovic and Zecevic, 1986; Arras, 1987; Yamaguchi et al., 1989; Carpenter, 1991; Voogd, 2003; O’Rahilly and Müller, 2006; Manto, 2010; Ristanovic et al., 2010). Most of these studies were confined to the dentate nucleus and did not provide the nowadays required resolution and histologic preparation standards (e.g., shrinkage correction). The most accurate histological post mortem data are based on 100 human cerebella (age range 22–72 years) with a histological sections thickness of 0.5 mm (Kozlova, 1984). Albeit only maxima of the *x*, *y*, *z* extension had been reported this data allowed to roughly estimate the volume of each cerebellar nucleus. Diedrichsen et al. (2011) provided MR-based volume data of the cerebellar nuclei and additionally computed the mean of the *x*, *y*, and *z* maxima, allowing an indirect comparison with Kozlova’s (1984) data. The aim of the present study was to map the cerebellar nuclei in histological sections of 10 *post mortem* brains to create cytoarchitectonic 3D probability maps in a standard reference space and to evaluate anatomical and functional partition of the cerebellar nuclei. Therefore we integrated the computed maps in the SPM Anatomy Toolbox (Eickhoff et al., 2005), and then used the respective representations for meta-analytic connectivity modeling as well as functional decoding (Eickhoff et al., 2012). Consequently we achieved a cytoarchitectonically based representation of the cerebellar nuclei in 3D space, and assigned its corresponding function by meta-analytic connectivity modeling.

## Materials and Methods

### Histological Techniques

We investigated 10 human *post mortem* brains (male/female: 5/5, age 58.7 ± 17.3 years, range 30–85 years; cf. **Table 1**) collected through the body donor program of the University of Düsseldorf (Germany) in accordance to local legal and ethical requirements. Subjects had no known history of neurological or psychiatric diseases. Details of the histological processing have been previously described in detail (e.g., Amunts et al., 1999). In short, brains were fixed for several months in 4% formalin or Bodian fixative. During fixation, the brains were suspended on the basilar artery to avoid compression or distortions. T1-weighted MRI scans [1.5T Siemens Magnetom SP scanner, 3D fast low angle shot (3D FLASH) pulse sequence, flip angle = 40∘, TR = 40 ms, TE = 5 ms, voxel size = 1 mm × 1 mm × 1.17 mm] were obtained to get a shape reference for further 3D-reconstruction of the histological sections. Artifacts (e.g., shrinkage of the brain, embedding in paraffin, and distortion of the sections due to cutting) could be eliminated in the reconstructed volume by matching it with the MR volume of the same brain using linear and non-linear correction procedures (cf. Homke et al., 2009). Following dehydration and embedding in paraffin, the brains were sectioned (20 μm). Nine coronal and one horizontal series of sections were analyzed. The sections were mounted on gelatin-coated glass slides, and stained for cell bodies with a modified silver method (Merker, 1983). On digital images of every 60th section the region of interest was marked and captured using a light microscope (Zeiss). The contours of the nuclei were drawn in serial section of both hemispheres, using high-resolution images (20 μm) of histological sections. Therefore, every 15th section was scanned with a flatbed scanner. The identification of the nuclei was done in accordance to criteria described in previous studies (e.g., Stilling, 1864; Weidenreich, 1899; Jakob, 1928; Carpenter, 1991; Nieuwenhuys et al., 2008).

**Table 1:** Sample of *post mortem* brains used for cytoarchitectonic analysis.

ID	Age	Sex	Shrinkage factor	Brain weight (*g*)	Cause of death
5	59	Female	2.15	1142	Cardio-respiratory insufficiency
6	54	Male	2.50	1757	Myocardial infarct
7	37	Male	2.25	1437	Heart failure
8	72	Female	1.90	1216	Renal failure
9	79	Female	1.51	1110	Heart failure
10	85	Female	1.72	1046	Mesenteric artery infarction
11	74	Male	2.20	1381	Cardiac infarction
12	43	Female	2.14	1198	Cardio-respiratory insufficiency
15	54	Male	1.60	1260	Accident
21	30	Male	1.84	1409	Morbus Hodgkin

### Volumetric Analysis of the Cerebellar Nuclei

The volumes of the nuclei were measured as previously described (Amunts et al., 2007). They were normalized and expressed as the fraction of the individual total brain volume in order to account for individual differences in total brain volume. The volumes were tested for sex and interhemispheric differences, as well as their interaction using pairwise permutation tests (*p* < 0.05; false discovery rate (FDR) corrected for multiple comparisons).

### Analysis of the Subdivision of the Dentate Nucleus

Cell densities of the dorsal and ventral parts of the dentate nucleus were measured in order to analyze differences between both parts. Therefore, the marked regions of interest on images of histological sections were obtained using a CCD-Camera (Axiocam MRm, ZEISS, Germany), which was connected to a light microscope (Axioplan 2 imaging, ZEISS, Germany) and operated by the Zeiss image analysis software Axiovision (4.8.0). Three sections per structure, hemisphere and brain were analyzed. Cell densities where measured using parts of the Grey Level Index (GLI) calculation pipeline to estimate the volume fraction of cell bodies (Wree et al., 1982; Schleicher and Zilles, 1990; Schleicher et al., 1999).

Therefore we delineated the dentate nucleus into a ventral and a dorsal partition using ImageJ ^1^. In a next step, the density of cells of each part was measured using in-house software based on MATLAB 8.1 ^2^. Both parts differed in the distribution, pattern, and morphology of neurons (see Results), resulting in a clear-cut border. Cell bodies were segmented in order to calculate binary- images (Schleicher and Zilles, 1990; Schleicher et al., 1999), and to measure the density of cells of each part using in-house software based on MATLAB 8.1^2^. Subsequently we calculated a quotient from the area of the segmented cells and the area of the whole structure [cell area (μm^3^)]/[structure area (μm^3^)] and compared the mean values between the two parts and hemispheres. Differences in cell density between the dorsal and ventral dentate nucleus were assessed using the non-parametric Wilcoxon-Sign-Rank test (*p* < 0.05, Bonferroni-corrected for multiple comparisons).

In addition, a Folding Index (FI) was estimated to quantify putative differences between a micro- and macrogyric aspect, which has previously been described (cf. Winkler, 1926; Voogd, 2003). These studies suggested that the dorsal part matches with the description of a microgyric part, while the ventral represents a macrogyric part. The FI is comparable to the Gyrification Index (Zilles et al., 1988, 2013), but estimates the gyrification of nuclei instead of the whole brain. In a first step, the contour of the dentate nucleus was labeled in images of 10 histological sections per hemisphere. In a second step, a convex hull representing the outer border of the dentate nucleus was drawn. The FI was then calculated as the ratio of these two measurements, i.e., FI = [Length (whole contour)]/[Length (hull contour)].

### Analysis of the Subdivision Generation of Probability Maps and 3D Reconstruction

The delineated nuclei were 3D-reconstructed in each *post mortem* brain, and then normalized to the single subject reference template of the Montreal Neurological Institute to a resolution of 1 mm × 1 mm × 1 mm (stereotaxic MNI-Colin27; Collins et al., 1994; Holmes et al., 1998; Evans et al., 2012). In addition, a manual segmentation of the cerebellum was performed using the ITK Snap software (Yushkevich et al., 2006) to improve the registration of the cerebellum. Superimposing the individual maps of each nucleus across brains then, yielded a probabilistic map, indicating how likely each nucleus was found at each voxel of the stereotaxic MNI-Colin27 template space.

### Mapping Function and Connectivity of the Delineated Nuclei

Functional interactions during task performance, in the context of neuroimaging experiments, i.e., co-activations, of the cerebellar nuclei were identified by meta-analytic connectivity modeling (Eickhoff et al., 2012) using the BrainMap database ^3^ (Fox and Lancaster, 2002; Laird et al., 2009, 2011). From this database, only mapping experiments in healthy subjects were considered, which yielded approximately 7.500 experiments at the time of analysis. Among these, all experiments with at least one peak activation coordinate within cytoarchitectonically defined seed regions were identified. The number of contributing studies was marginal for the ventral dentate nucleus (VDN) and dorsal dentate nucleus (DDN) separately or the emboliform and globose nuclei. Accordingly only minor effects occurred for probing these subregions. Therefore, the maximum probability map representations of the cytoarchitectonically defined entire dentate nucleus and interposed nucleus in stereotaxic MNI-Colin27 space were used as seed regions (Eickhoff et al., 2006). Across these, an Activation Likelihood Estimation meta-analysis (Eickhoff et al., 2012; Turkeltaub et al., 2012) was conducted in order to identify areas of converging activity across these experiments. Evidently, the highest convergence between studies occurs within the seed (as all included experiments were selected based upon co-activity with the seed region). In comparison, significant (*p* < 0.05) convergence in areas beyond the seed is indicative of consistent co-activation (i.e., functional connectivity) with the seed region. The resulting statistically thresholded co-activation map (*p* < 0.05, cluster-level family wise error (FWE) corrected for multiple comparisons) thus provided the results of the meta-analytic connectivity modeling analysis.

The functional characterization of the cerebellar nuclei was based on the meta-data available for each neuroimaging experiment included in the BrainMap database. Functional profiles were determined by identifying taxonomic labels, for which the probability of finding activation in the respective region was significantly (*p* < 0.05) higher than by chance. Significance was established using a binomial test (*p* < 0.05, corrected for multiple comparisons; Cieslik et al., 2013; Clos et al., 2013).

## Results

### Cytoarchitecture of the Cerebellar Nuclei

**FIGURE 1:**
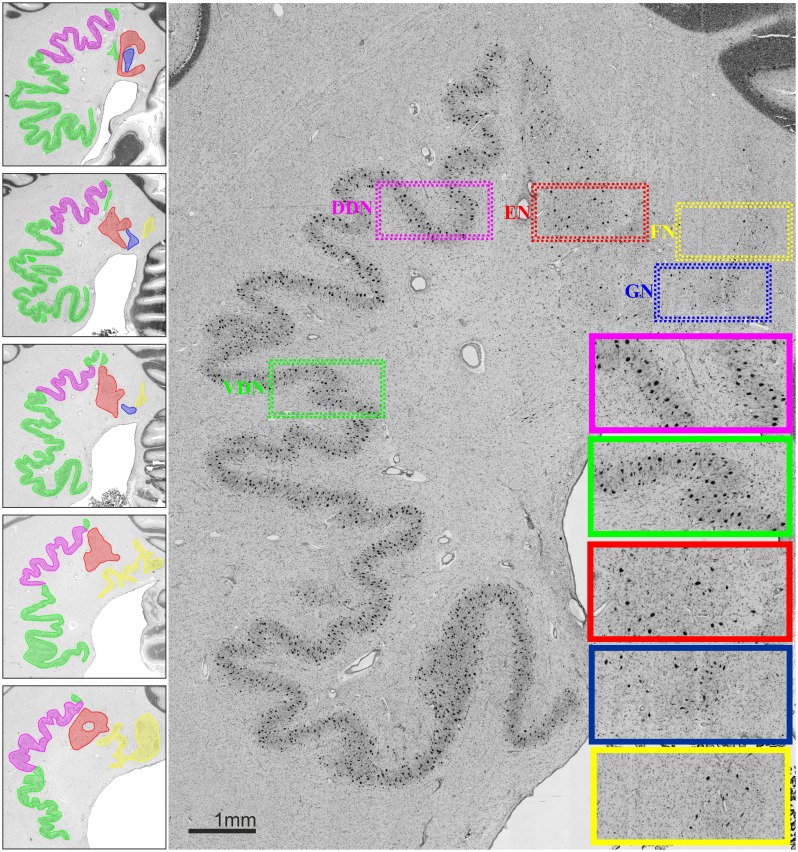
**(Left)** Localization and extent of cerebellar nuclei in a rostro-caudal sequence of histological sections of a post mortem brain; distance between sections 60 μm. **(Right)** Cytoarchitecture of each cerebellar nucleus and the two parts of the dentate nucleus. Magenta: dorsal dentate nucleus (DDN); green: ventral dentate nucleus (VDN); red: emboliform nucleus (EN); blue: globose nucleus (GN); yellow: fastigial nucleus (FN).

**FIGURE 2:**
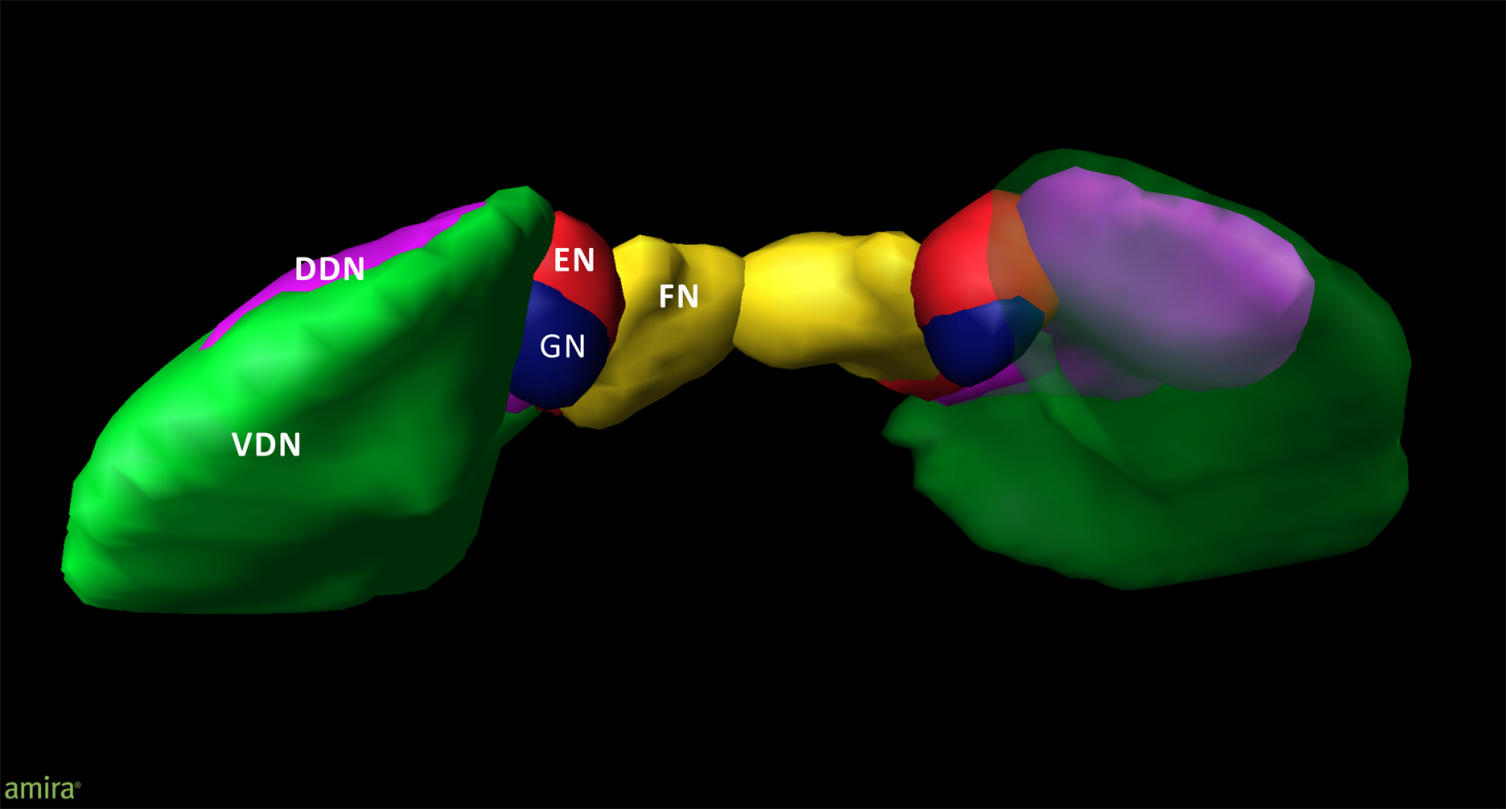
**3D model of the deep cerebellar nuclei (posterior to anterior view) of an individual brain (post mortem brain10);** visualization by Amira 5.6.0 (www.amira.com). Dorsal dentate nucleus (DDN; magenta); ventral dentate nucleus (VDN; green); emboliform nucleus (EN; red); globose nucleus (GN; blue); fastigial nucleus (FN; yellow). Due to the smoothing, the dentate appears less denticulated than it is. The transparency of the right ventral dentate nucleus clarifies the partly covered extend of the DDN.

An overview of the cytoarchitectonic features of the four cerebellar nuclei including the subdivision of the dentate nucleus is provided in **Figure 1**. **Figure 2** shows a 3D representation of the cerebellar nuclei to illustrate the intern-relationship between the delineated structures.

**FIGURE 3:**
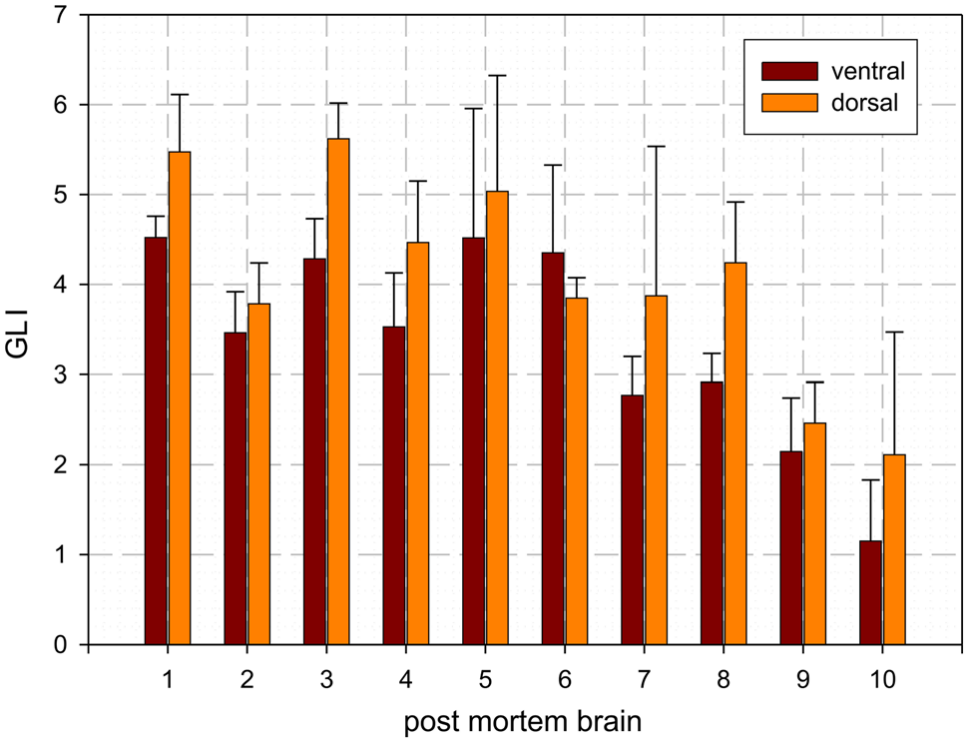
**Mean values and SD of cell density distribution of the ventral and dorsal dentate nucleus as estimated by the Grey Level Index (GLI): [cell area (μm^**2**^)]/[structure area (μm^**2**^)]**.

*The dentate nucleus* is the largest and most lateral cerebellar nucleus. It consisted of densely packed rounded multipolar neurons. Although there was a mixture of cell sizes within the dentate nucleus, large cells were predominant. The dentate nucleus appeared as a convoluted band with its hilus located medially. Based on local differences in cell density and size, the dentate nucleus could be microscopically subdivided in a dorsal and ventral part by a clear-cut border, whereby the dorsal part had a significantly higher cell density than the ventral one. The mean Grey Level Index values, estimating cell density observer-independently (Wree et al., 1982; Schleicher and Zilles, 1990; Schleicher et al., 1999), and the corresponding SDs were as follows: left dorsal: 4.09 ± 0.78; right dorsal 4.09 ± 0.74; left ventral: 3.36 ± 0.62; right ventral: 3.45 ± 0.65 (cf. **Figure 3**). Differences between dorsal and ventral parts were significant (*p* < 0.05), whereas left–right differences did not reach significance (*p* > 0.05).

**FIGURE 4:**
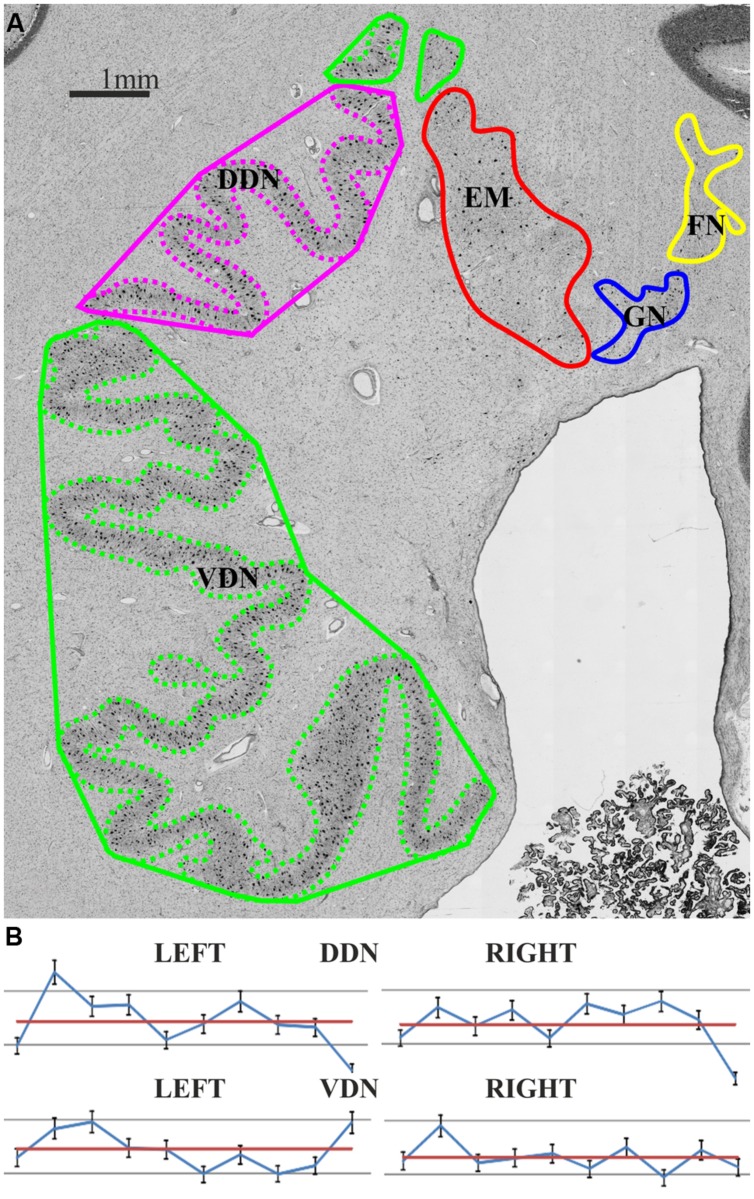
**(A)** The Folding Index (FI) provides information about the gyrification and was estimated as the quotient of Length (whole contour: dashed line) per Length (hull contour: solid line). Magenta: dorsal dentate nucleus (DDN); green: ventral dentate nucleus (VDN); red: emboliform nucleus (EN); blue: globose nucleus (GN); yellow: fastigial nucleus (FN); **(B)** Individual FI values of each partition and hemisphere of 10 post mortem brains (blue: FI; red: mean FI).

In contrast, no differences were observed with respect to the FI, which was nearly identical between both parts (FI dorsal average of left and right = 1.69 ± 0.57; ventral average of left and right = 1.69 ± 0.62; cf. **Figure 4**).

*The emboliform nucleus* was positioned close to the vermis, and next to the dentate hilus in all 10 brains. In comparison to the dentate nucleus, it was formed by less densely packed large neurons (**Figure 1**).

*The globose nucleus* was also located close to the vermis, between the emboliform and the fastigial nucleus. It was the smallest of the four cerebellar nuclei. In all investigated brains, its neurons were small and more densely packed as compared to those in the emboliform nucleus (**Figure 1**). As previously described (e.g., Kozlova, 1984), its shape did not follow the name “globose,” as it often appeared variably elongated.

*The fastigial nucleus* was the most medially located cerebellar nucleus, located in close vicinity of the fourth ventricle. Starting at its lateral border, tentacle-like bands of more spikey cells were visible that spread to the lateral border of the vestibular nucleus.

#### Volumetric Analysis of the Cerebellar Nuclei

There were no statistically significant effects (*p* < 0.05) of sexes or hemisphere on the volumes of any of the delineated nuclei (**Table 2**). Bilateral mean values for each cerebellar nucleus and their SD are shown in **Table 2**. The dentate nucleus was the largest cerebellar nucleus, with its dorsal part being about three times smaller than the ventral one. Nevertheless, this (smaller) dorsal part of the dentate nucleus was still about two times larger than the emboliform and the fastigial nuclei. The globose nucleus as the smallest cerebellar nucleus comprised only approximately a fifth part of the volume of the emboliform nucleus.

**Table 2:** Mean volumes (mm^3^) and SDs (in brackets) of each cerebellar nucleus of grouped hemispheres and sexes were calculated from the shrinkage corrected volumes of 10 post mortem brains. Male/female volumes represent the mean volumes of left and right hemisphere volumes. [(pair wise permutation tests; no differences with *p* < 0.05); DN: dentate nucleus; DDN: dorsal dentate nucleus; VDN: ventral dentate nucleus; IN: interposed nucleus; EN: emboliform nucleus; GN: globose nucleus; FN: fastigial nucleus] supplemented by MRI volume data ^**1**^Diedrichsen et al. (2011).

	Post mortem					MRI^1^	
	Right	Left	Male	Female	Bilateral	Right	Left
DN	394.5 (94.5)	390.2 (99.3)	433.2 (104.9)	351.5 (75.6)	784.7 (192.7)	366.1 (85.2)	362.8 (89.2)
DDN	93.5 (46.2)	88.7 (42.1)	94.9 (49.6)	87.2 (43.2)	182.1 (88.1)	–	–
VDN	301.0 (61.3)	301.5 (67.9)	338.3 (57.8)	264.3 (48.6)	602.5 (127.3)	–	–
IN	59.8 (12.2)	59.0 (11.9)	61.2 (9.1)	57.6 (14.8)	118.7 (23.5)	36.1 (11.4)	35.9 (14.2)
EN	50.2 (12.4)	49.5 (12.2)	50.4 (12.3)	49.3 (13.1)	99.7 (24.0)	–	–
GN	9.5 (4.0)	9.5 (4.7)	10.8 (5.3)	8.3 (3.1)	19.0 (8.5)	–	–
FN	45.0 (8.5)	46.4 (13.4)	50.8 (11.6)	40.5 (5.8)	91.4 (20.4)	8.2 (5.2)	9.2 (5.2)

#### Probabilistic Maps of the Cerebellar Nuclei

**FIGURE 5:**
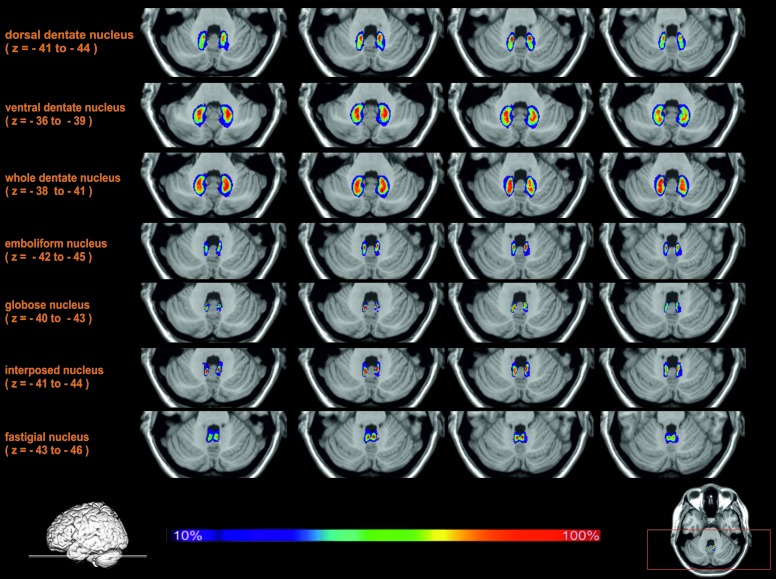
**Exemplary transversal sections through the stereotaxic MNI-Colin27 reference brain with probability maps of the cerebellar nuclei**. The maximal overlap in each nucleus was 100% (shown in red). Regions with lower probabilities correspond to a higher intersubject variability and are shown in blue and green colors.

All delineated structures were spatially normalized to the stereotaxic MNI-Colin27 single subject template and then combined across subjects to calculate probabilistic maps of cerebellar nuclei in stereotaxic space. In correspondence to the localization of the nuclei in each individual brain, all nuclei were located in the depth of the cerebellar white matter and showed the expected relative position (laterally: dentate nucleus; paravermal: first emboliform, then globose nucleus; medial: fastigial nucleus). The interindividual variability of the nuclei was low (**Figure 5**). There was only a relatively moderate overlap between the probabilistic maps of neighboring nuclei. The probabilistic maps were used to analyze co-activation patterns in order to characterize their involvement into different cognitive functions.

### Whole-Brain Co-activation Patterns of the Cerebellar Nuclei

**FIGURE 6:**
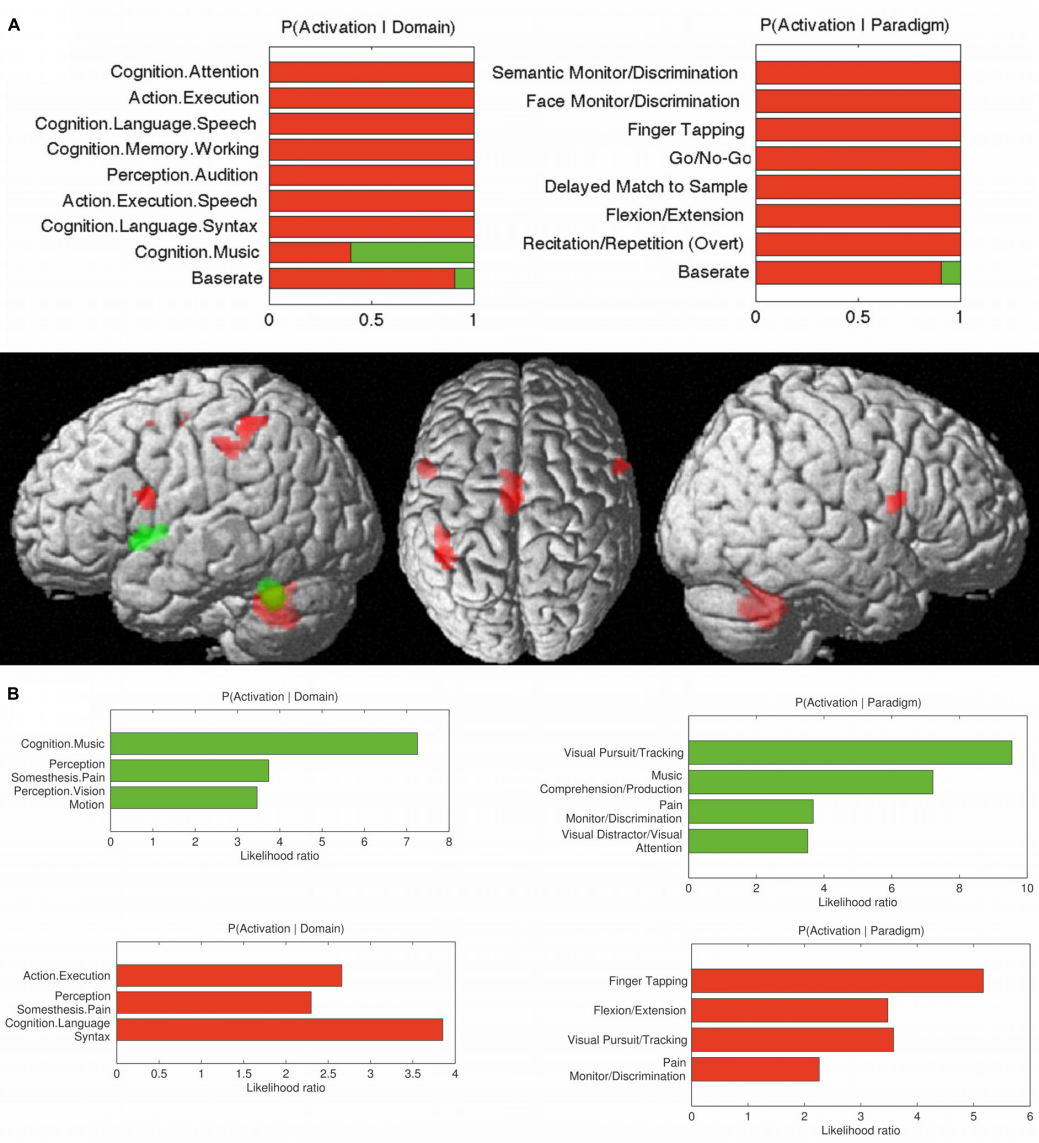
**(A)** Contrasts in behavioral domains between cerebellar interposed (green) and the dentate (red) nuclei (*p*FDR < 0.05); **(B)** Behavioral domain information for the cerebellar interposed (green) and the dentate (red) nuclei (*p*FDR < 0.05).

Co-activation mapping and functional decoding for the combined dentate (ventral and dorsal) and interposed (emboliform and globose) nuclei were performed. For these analyses, the regions of interest were defined by the maximum probability map representations of the respective histologically defined nuclei in stereotaxic MNI-Colin27 space (Eickhoff et al., 2006). For the dentate nucleus, we found significant (*p* < 0.05, corrected for multiple comparisons) co-activations with thalamus, supplementary motor area (SMA) and putamen as well as within area 44 (Amunts et al., 1999), superior parietal area 7PC (Scheperjans et al., 2008a), inferior parietal area PFt (Caspers et al., 2006), and the superior frontal gyrus (SFG). The interposed nucleus likewise showed, though more limited, co-activation with the putamen, SMA, area 44 and the SFG (cf. **Table 3**). Directly compared with the dentate nucleus (cf. **Figure 6**), the interposed nucleus showed a significantly (*p* < 0.05) higher connectivity with the left insular lobe [stereotaxic MNI-Colin27: (-40, 0, 2), cluster size: 104 mm^3^] and the left thalamus [stereotaxic MNI-Colin27: (-10, -18, 8); cluster size: 90 mm^3^]. In turn, the dentate nucleus showed higher connectivity with left area 6 [SMA; stereotaxic MNI-Colin27: (-4, -14, 54); cluster size: 215 mm^3^], the left inferior parietal lobe [Pft; stereotaxic MNI-Colin27: (-46, -40, 56); cluster size: 138 mm^3^], and the inferior frontal gyrus [area 44, stereotaxic MNI-Colin27: (-58, 8, 18); cluster size: 56 mm^3^].

**Table 3:** Co-activation clusters for the cerebellar nuclei.

	Cluster Size	Z	Stereotaxic MNI-Colin27			Anatomic Localization (probabilistic anatomical location)
			*x*	*y*	*z*	
Dentate nucleus^∗^	2163	6.94	-34	+22	+4	Left anterior Insula Lobe
		6.26	-26	+18	-4	Left Medial Putamen
		6.09	-52	+6	+38	Left Precentral Gyrus (BA 44^1^)
		5.84	-46	+4	+8	Left Rolandic Operculum
		5.78	-48	+6	+6	Left Inferior Frontal Gyrus
		5.49	-54	+8	+22	Left Inferior Frontal Gyrus (BA 44^1^)
	1701	8.32	-2	+2	+54	Left SMA
		4.55	+6	+18	+46	Right SMA
		4.29	+20	+0	+58	Right Superior Frontal Gyrus
	646	8.31	+38	+20	-2	Right Anterior Insula Lobe
		5.01	+36	-4	+2	Right Putamen
		4.34	+28	+10	-4	Right Putamen
		4.18	+24	-2	+2	Right Pallidum
	538	8.32	-12	+20	+4	Left Thalamus
		8.31	-14	+14	+6	Left Thalamus
		4.18	-20	-16	+0	Left Thalamus
	516	5.86	+58	+10	+24	Right Inferior Frontal Gyrus (BA 44^1^)
		5.50	+58	+8	+10	Right Rolandic Operculum (BA 44^1^)
	420	5.72	-42	-48	+50	Left Inferior Parietal Lobe (7PC^2^) Left Inferior Parietal Lobule (PFt^3^)
		5.54	-30	-50	+50	Left Inferior Parietal Lobule (7PC^2^)
		5.50	+58	+8	+10	Right Rolandic Operculum (BA 44^1^)
Interposed nucleus^∗^	640	5.49	+20	+0	+58	Right Superior Frontal Gyrus
		5.41	+0	+10	+5	Left SMA
		4.56	-4	+22	+44	Left SMA
		4.44	+8	+4	+60	Left SMA
		3.81	+12	+14	+40	Right SMA
		3.28	-4	+2	+60	Left SMA (BA 6^4^)
	307	7.99	-14	-14	+10	Left Thalamus
	238	5.53	+28	+10	-4	Right Putamen
		4.10	+40	+18	-4	Right Anterior Insula Lobe
		3.99	+38	+16	-6	Right Anterior Insula Lobe
	266	4.65	-48	+8	+2	Left Rolandic Operculum (BA 44^1^)
		4.51	-44	+14	-4	Left Anterior Insula Lobe
		3.78	-44	+0	+2	Left Insula Lobe
		3.45	-44	+24	-4	Left Inferior Frontal Gyrus
	243	5.01	-32	+16	+8	Left Insula Lobe
		4.65	-22	+6	+2	Left Putamen

Macroanatomic localization with respect to gyri, sulci, and major subcortical nuclei; localization with respect to cytoarchitectonic probabilistic maps if available. *p_FDR_ < 0.05. ^1^Amunts et al. (1999), ^2^Scheperjans et al. (2008b), ^3^Caspers et al. (2008), ^4^Geyer (2004).

The behavioral domains and paradigm classes significantly (*p* < 0.05) associated with the dentate and interposed nuclei are illustrated in **Figure 6**. Both structures were found to be activated by pain. In addition, the interposed nucleus (green) was significantly (*p* < 0.05) associated with music comprehension and visual perception. In turn, the behavioral domains and paradigm classes of the dentate nucleus (red) comprised cognitive, speech, and in particular motor related functions.

## Discussion

Cerebellar nuclei have a strategic position by representing the almost unique source of output within the cerebellar circuitry (Manto and Oulad Ben Taib, 2010). This study presents cytoarchitectonically based 3D probability maps of the human cerebellar nuclei including their application to study their function and functional connectivity. Besides providing information on the cytoarchitectonic characteristics and precise anatomical localization of each nucleus, the current study also subdivided the dentate nucleus into a ventral and a dorsal part based on cytoarchitectonic criteria. These maps of the cerebellar nuclei in the stereotaxic MNI-Colin27 reference space are available to the scientific community ^4^, and may facilitate interpretation of *in vivo* structural and functional imaging data with respect to the microstructural correlates. We here employed these maps to investigate task-based functional connectivity of the cerebellar nuclei using meta-analytic co-activation mapping and to perform a quantitative functional characterization.

### Mapping Results

In this study, the borders of the cerebellar nuclei, were delineated in 10 *post mortem* brains based on cytoarchitectonic differences, and stereotaxic maps were calculated (**Table 2** for comparison of the *post mortem* and recent MRI data: Diedrichsen et al., 2011). The current volume of the dentate nucleus is nearly identical to that reported based on MRI measurements. Small differences between both estimates may be caused by partial volume effects, which are more relevant in lower resolution MR images. Three other previous MRI studies provided substantially larger volumes (840 mm^3^: Dimitrova et al., 2002, 2006; 900 mm^3^: Deoni and Catani, 2007). They may overestimate the true volume, caused by the complex shape of the dentate with its large surface area. Finally, an older histological estimate yielded a much lower volume of the dentate nucleus, but no shrinkage correction was applied (155 mm^3^: Höpker, 1951). Still, the volume would be considerably smaller than that of the present study. The volume of the interposed nucleus of the present study was slightly larger than previously estimated by MRI (Diedrichsen et al., 2011). The volume of the fastigial nucleus in the Diedrichsen et al. (2011) MRI atlas finally seemed to be underestimated relative to the current *post mortem* results, but also in comparison to earlier histological data (Dejerine and Dejerine-Klumpke, 1901; Jakob, 1928; Jansen et al., 1958).

To the best of our knowledge, no previous volumetric data has been presented for the ventral and dorsal subdivisions of the dentate nucleus and the subdivided interposed (globose and emboliform) nucleus. While the small size of these structures is still a major challenge for MRI based delineation *in vivo*, ultra-high field MRI with high resolution may allow an even better delineation in future (Forstmann et al., 2012). In summary, the estimated volumes for all cerebellar nuclei differ to some degree between studies of *in vivo* and *post mortem* approaches.

Although a comparison of volume data for the subdivision of dentate nucleus is currently not available, we will here contrast the current *post mortem* data with some other methods and studies. A significantly different cell density distribution between the ventral and dorsal part of the dentate nucleus, with the latter featuring a higher cell density and bigger cells, is in accordance with previous reports (Arras, 1987; Dum et al., 2002; Voogd, 2003; Timmann, 2012). Albeit transitional areas were reported (Arras, 1987), the present observation revealed a clear-cut border between the ventral and the dentate nucleus. Interestingly, the reported volume differences between these two parts of the dentate nucleus, with the ventral part being about three times larger, may relate to evolutionary development.

The larger size of the human ventral dentate nucleus may reflect the general evolutionary trend of “neocorticalization” and the marked development of higher motor functions and ultimately cognition in the primate lineage (Fix and Treff, 1970). Accordingly, the (larger) ventral part of the dentate nucleus has been termed “neo-dentate” (cf. Weidenreich, 1899). Moreover, the embryogenetic differentiation of the ventral dentate nucleus developed to the same time as the cerebellar hemispheres, while development of the dorsal part coincided with that of the vermal parts and the anterior lobe of the cerebellum (Murofushi, 1974). From a different angle, it has been shown that in case of neocerebellar atrophy the dorsal dentate nucleus remains untapped (Brun, 1917). Moreover, the number of interneurons is higher in the parvocellular – ventral – part of the dentate nucleus (Arras, 1987), which has been interpreted as a developmental adaptation, described similarly for the isocortex (Schlegelberger and Braak, 1982). In summary, there is thus converging evidence for a dorsal–ventral distinction of the human dentate nucleus in which the larger ventral part has co-evolved with the cerebral cortex (cf. “neocorticalization” Fix and Treff, 1970) and is related to higher cognitive-motor functions. Morphological differences have long been discussed as another aspect of such differentiation (Jansen et al., 1958; Fix and Treff, 1970; Arras, 1987). Summarized, the magnocellular dorsal part has been described as microgyric and the parvocellular ventral part as macrogyric (e.g., Voogd, 2003). In contrast to previous reports on a macro- and microgyric part within the dentate nucleus (Winkler, 1926; Voogd, 2003) no differences were found with respect to the FI as a measure of “gyrification.” This finding, in turn, is in accordance with another more recent study, where a gyrification difference within the dentate was only found in macaques but not in human brains (Sultan et al., 2010). Finally it should be mentioned, that the literature provides evidence for a more subtle and somatotopic distinction of the cerebellar cortex (Hampson et al., 1946; Snider and Eldred, 1952; Grodd et al., 2001). It can therefore be hypothesized that this may also apply to the cerebellar nuclei, which are interconnected with the different parts of the cerebellar cortex. Arras (1987) reported a transitional area between the ventral and dorsal dentate nucleus. Results of the present observation did not support this assumption, and no differences in cytoarchitecture have been observed in-between the dorsal and the ventral parts. Other studies point toward a somatotopic organization of the dentate nucleus in human (*in vivo*) and monkeys (tracer studies; Dum et al., 2002; Dum and Strick, 2003; Kuper et al., 2013). Dum et al. (2002) used tracer injections into the primary motor cortex to provide evidence for somatotopically organized connectivity patterns in the dorsal dentate nucleus (from rostral to caudal: arm, leg, and face), and somatotopic connectivity with the premotor cortex and in the middle third of the caudate. A third somatotopically organized pattern of connections to prefrontal areas 46, 9, and 7 was observed in the ventral dentate nucleus. In the present study we did not find consistent cytoarchitectonic evidence for further subdivisions of the dentate nucleus. This, however, does not rule out potential distinctions that may emerge from, e.g., myeloarchitecture or multi-receptor mapping.

### Co-activation Patterns of the Cerebellar Nuclei

Recent studies showed that the cortex of the cerebellar hemispheres, in particular Crus I and Crus II (lobolus VIIA; cf. Schmahmann et al., 1999) is strongly involved in cognitive functioning (Kelly and Strick, 2003; Balsters et al., 2013; see also Tomlinson et al., 2013 for an overview). These structures, in turn, are linked to the ventral dentate nuclei as described above (Voogd, 1964; Rossum, 1969). In line with this model and non-human approaches (e.g., Strick et al., 2009), the present study showed the dentate nucleus to be engaged in motor-related and cognitive processes. While single Tracer studies (Dum et al., 2002) distinguished a ventral and a dorsal part of the dentate nucleus, the meta-analysis shown here represents the functional associations and connections of the entire dentate nucleus, due to the limited number of contributing studies when analyzing subdivisions. Nevertheless, we found the dentate nucleus involved in basal executive as well as in higher order motor and cognitive functions. The results of our analysis on the entire dentate nucleus are in line with this focused investigation and primate data. In addition, the functional decoding not only showed an involvement of the dentate nucleus in cognitive and motor tasks, but also with respect to pain processing. Cerebellar involvement in pain-perception has been described earlier (Glickstein, 2007; Timmann and Daum, 2007; Strick et al., 2009). In general, the delineated functional connectivity of the dentate nucleus matches well with reports from invasive approaches dealing with structural connectivity mapping in non-human primates (see a review by Dum et al., 2002), even though no interactions with the primary motor cortex was observed in our findings. We did, however, observe significant (*p* < 0.05) co-activation between the dentate nucleus and the SMA. This finding matches with previous descriptions, which show that neurons from the dorsal “motor” domain of the dentate nucleus in monkey brains project to the SMA (Akkal et al., 2007). The co-activations of the dentate nucleus with the inferior and anterior parietal cortex are in line with tracing data revealing a connection between the parietal cortex and the (ventral) dentate nucleus (Dum et al., 2002). While there is no primate data to this end, the link between the dentate nucleus and speech as well as its co-activation with left BA 44 is in good agreement with a previous fMRI study (Thurling et al., 2012). Finally, it has been argued, that a particular function of the cerebellar hemisphere, which remits its output throughout the dentate nucleus, is rhythm perception and memory (Jerde et al., 2011; Pecenka et al., 2013). The current finding of an association between the dentate nucleus and music comprehension supports this view.

Only a small number of previous studies have reported on anatomy, function and connectivity of the interposed nuclei, most likely due to difficulties in the precise localization of these small structures. It has been reported that the paravermal interposed nuclei may be related to associative motor learning, i.e., eye blink reflex (Gerwig et al., 2003; Parker et al., 2009). The present study found that the interposed nuclei are associated with visual perception and attention as well as visuomotor tasks, which would be in line with these previous findings. Likewise, the association to somesthetic domain resonates well with older accounts which postulated a role for the interposed nuclei in (disturbed) sensory perception and cerebellar tremor (Vilis and Hore, 1977). Like the dentate nucleus, also the interposed nucleus features co-activations with the SMA. The SMA represents a key structure for bimanual movement coordination and reach-to-grasp functions (Wilson et al., 2014) and there is also strong evidence from human and monkey studies that the interposed nucleus plays an important role for reaching-to-grasp movements (van Kan et al., 1994; Monzee and Smith, 2004; Kuper et al., 2011b). Given that, we would thus argue, that these interactions may play a particular role in the cortico-cerebellar tuning of complex, coordinated arm, and hand movements.

## Conclusion

We here reported on the first probabilistic atlas of the human cerebellar nuclei based on a cytoarchitectonic histological examination in 10 *post mortem* brains. The probabilistic maps in the stereotaxic MNI-Colin27 space provide new opportunities to relate structure, function, and dysfunction of the cerebellar nuclei as obtained in the living human brain to microscopically defined nuclei. To foster their use, the proposed maps will be integrated into the JuBrain atlas and freely distributed as part of the SPM Anatomy Toolbox ^5^.

## Author Contributions

ST performed the cytoarchitectonic mapping, interpretation of data and wrote the first draft of the manuscript.

SB contributed to the development of methods for parcellation and analysis and revised the manuscript.

SE contributed to the meta-analytic connectivity modeling analysis and revised the manuscript.

HM contributed to the 3D reconstruction of the postmortem brains, their transformation into the stereotaxic MNI-Colin27 space and the computation of the probabilistic maps.

MM contributed to interpretation of data for the work and revised the manuscript.

KA contributed to the design of the study, the development of methods for parcellation and analysis, the interpretation of results and writing the manuscript.

All authors have approved the final version of the work to be published and agree to be accountable for all aspects of the work in ensuring that questions related to the accuracy or integrity of any part of the work are appropriately investigated and resolved.

## Conflict of Interest Statement

The authors declare that the research was conducted in the absence of any commercial or financial relationships that could be construed as a potential conflict of interest.

## Footnotes

1 http://imagej.nih.gov/ij/

2 http://mathworks.com

3 www.brainmap.org

4 www.fz-juelich.de/inm/inm-1/EN

5 http://www.fz-juelich.de/SharedDocs/Downloads/INM/INM-1/DE/Toolbox
